# Circular RNAs in the tumour microenvironment

**DOI:** 10.1186/s12943-019-1113-0

**Published:** 2020-01-14

**Authors:** Zhonghua Ma, You Shuai, Xiangyu Gao, Xianzi Wen, Jiafu Ji

**Affiliations:** 1 Key Laboratory of Carcinogenesis and Translational Research (Ministry of Education), Division of Gastrointestinal Cancer Translational Research Laboratory, Peking University Cancer Hospital & Institute, Beijing, People’s Republic of China; 2 Department of Gastrointestinal Surgery, Peking University Cancer Hospital, Beijing, People’s Republic of China; 3 Department of Medical Oncology, Jiangsu Cancer Hospital, Jiangsu Institute of Cancer Research, The Affiliated Cancer Hospital of Nanjing Medical University, Nanjing, Jiangsu People’s Republic of China

**Keywords:** Circular RNAs, Tumour microenvironment, Molecular mechanism, Biomarker, Target, Drug resistance

## Abstract

**Background:**

Circular RNAs (circRNAs) are a new class of endogenous non-coding RNAs (ncRNAs) widely expressed in eukaryotic cells. Mounting evidence has highlighted circRNAs as critical regulators of various tumours. More importantly, circRNAs have been revealed to recruit and reprogram key components involved in the tumour microenvironment (TME), and mediate various signaling pathways, thus affecting tumourigenesis, angiogenesis, immune response, and metastatic progression.

**Main body of the abstract:**

In this review, we briefly introduce the biogenesis, characteristics and classification of circRNAs, and describe various mechanistic models of circRNAs. Further, we provide the first systematic overview of the interplay between circRNAs and cellular/non-cellular counterparts of the TME and highlight the potential of circRNAs as prospective biomarkers or targets in cancer clinics. Finally, we discuss the biological mechanisms through which the circRNAs drive development of resistance, revealing the mystery of circRNAs in drug resistance of tumours.

**Short conclusion:**

Deep understanding the emerging role of circRNAs and their involvements in the TME may provide potential biomarkers and therapeutic targets for cancer patients. The combined targeting of circRNAs and co-activated components in the TME may achieve higher therapeutic efficiency and become a new mode of tumour therapy in the future.

## Background

Circular RNAs (circRNAs), a new class of endogenous non-coding RNAs (ncRNAs), were originally considered as non-functional by-products of aberrant splicing [1–3]. With the introduction of RNA-sequencing (RNA-seq) technology and bioinformatics, thousands of circRNAs are shown to be abundant in eukaryotic cells [2–5]. Moreover, the simultaneous use of prediction tools can benefit the unveiling of circRNAs, such as KNIFE, PTESFinder, MapSplice, CIRCexplorer and etc. [6]. Of note, miARma-Seq, a comprehensive pipeline analysis suite, is able to realize easy implementation of diverse algorithms [6]. Meanwhile, it is essential to perform accurate quantification of circRNAs. Specifically, circRNAs expression can be validated by real-time quantitative polymerse chain reaction (RT-qPCR), micro-drop digital PCR, northern blot and in situ hybridization (ISH) [6].

Despite there is a fall of interest for some years, circRNAs have been identified as important hallmarks of various tumours [7–10]. Of note, tumour cells are not alone, since the tumour microenvironment (TME), as a key determinant in all stages of cancer development and progression, is a complex ecosystem involving the coevolution of both cancerous cells and the surrounding stroma [11]. Multiple cellular components in the TME include immune cells (T-cells, tumour associated macrophages (TAM), dendritic cells, mast cells etc.), cancer-associated endothelial cells (CAEs), cancer-associated fibroblasts (CAFs) and cancer stem cells (CSCs) [12, 13]. Non-cellular counterparts consist of growth factors, cytokines, as well as extracellular matrix (ECM), which supplies not merely an inert place for this game [12, 13].

Currently, the circRNA-based communication within the TME has greatly attracted the scientific community. The clinical prospects of cancer therapy, targeting key counterparts of the TME, are encouraging [14, 15]. The establishment of circRNA-involved TME network may provide an opportunity for targeted therapy based on the interplay with circRNAs, facilitating the development of more effective therapeutics for various cancers [16, 17]. The most recent published reviews of circRNAs mainly focus on their biological roles in human cancers [18, 19]. However, the current knowledge of interplay between circRNAs and TME has not been systematically summarized to date. In this review, we document the biogenesis, characteristics, and mechanistic models of circRNAs in various cancers. Moreover, we provide the first overview of the interplay between circRNAs and cellular/non-cellular counterparts of the TME and highlight the potential of circRNAs as prospective biomarkers or targets in cancer clinics. Finally, we discuss the biological mechanisms through which the circRNAs drive development of resistance, revealing the mystery of circRNAs in drug resistance of tumours.

## Biogenesis, classification and characteristics of circRNAs

Early in 1976, circRNAs were firstly discovered in the Sendai virus and plant-infected viroids [5]. However, in the following decades, most circRNAs were considered as “splicing noise” or the by-products of RNA processing [3]. Despite various formations of circRNAs, most recently explored circRNAs are generated from precursor mRNAs (pre-mRNAs), where a downstream 5′splice donor site is linked to an upstream 3′ splice acceptor site [1, 2].

**Fig. 1:**
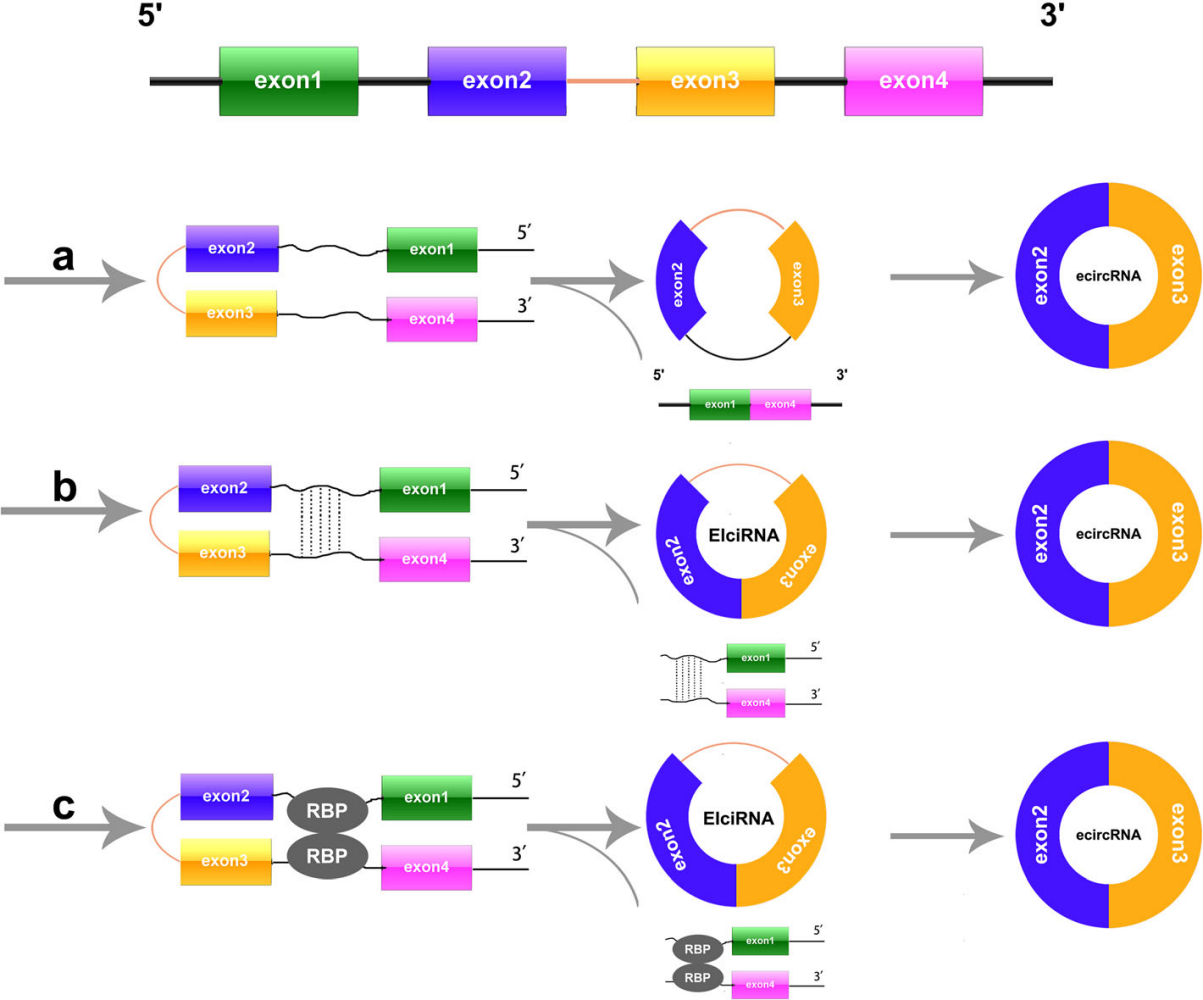
Biogenesis of ecircRNA and EIciRNA. **a** Exon skipping and formation of intra-lariat induced circulation. The splice donor in 3′ end of exon 1 and splice acceptor in 5′ end of exon 4 are covalently jointed together to form a RNA lariat containing skipped exons 2 and 3. Then, an ecircRNA were further formed by removing introns. **b**. Intron-pairing-induced circularization. The flanking introns form a circRNA by base pairing. Then, ecircRNA or ElciRNA are produced through removing or retaining introns. **c**. RBP-pairing-induced circularization. RBPs interact with the sequence motifs of the upstream and downstream introns to build a bridge between introns, thus facilitating the head-to-tail end-joining of exon2 and exon3. In the end, a circular RNA is produced

**Fig. 2:**
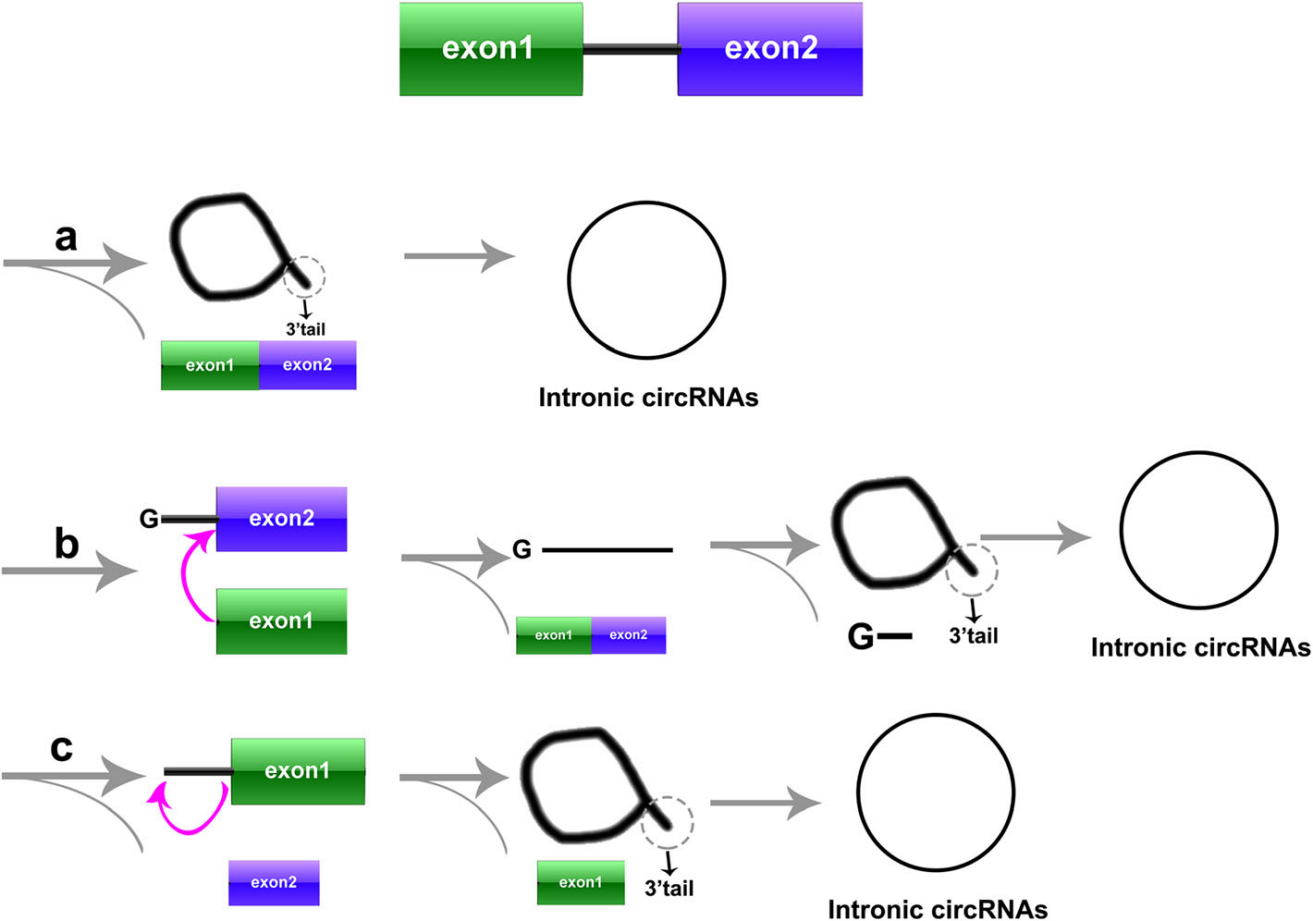
Biogenesis of circRNAs from intronic circRNAs. **a** Circular intron RNA (ciRNA). The circle formation requires prior release of exon 2. The 2′-OH group of the 3′-terminus attacks the phosphodiester bond near 5′-splice site of the intron, leading to formation of a circular RNA with 2′,5′-phosphodiester. **b**. Circular RNA from group I introns. An exogenous guanosine(G) attacks the 5′ terminus of the intron and exon 1 is cleaved due to transesterification. The 3′-hydroxyl of the free exon 1 acts as a nucleophile to attack the 5′-terminus of exon 2, producing a linear intron and the ligated exons. The 2′-hydroxyl group near the 3′ terminus of the linear intron attacks the phosphodiester bond near the 5′ terminus, forming a RNA lasso having a 2′,5′-phosphodiester. Finally, a short 3′ tail is released to form a circRNA from group I introns. **c**. Circular RNA from group II introns. Splicing the pre-mRNA to form a RNA lasso with a 2′,5′-phosphodiester. Then, a ciRNA is formed by removing the 3′ tail of the RNA lasso

With the advent and wide application of computational approaches and bioinformatics, the widespread expression of circRNAs has been uncovered in different cells among various species [20]. For example, Jeck et al. identified more than 25,000 circRNAs in cultured human fibroblasts [21]. Sebastian et al. detected approximately 2000 human, 1900 mouse and 700 nematode circRNAs from sequencing data [22]. Shortly after, Guo et al. developed a computational pipeline to expand identification of circRNAs from sequencing data [23]. Intriguingly, 7112 circRNAs was annotated from 39 biological samples with varied number of detectable circRNAs [23]. Based on the source of generation, circRNAs can be divided into four categories: exon circRNAs (ecircRNAs), intron circRNAs, exon-intron circRNAs (EIciRNAs), and intergenic circRNAs [24]. The biogenesis and classification of circRNAs are shown in Fig. 1 and Fig. 2. (1) More than 80% of circRNAs are ecircRNAs that contain only 3′ → 5′ linked exon sequences [25]. (2) Intron circRNA is a generic term for a class of circRNAs including circular intron RNAs (ciRNAs), excised group I introns, excised group II introns, excised tRNA introns and circRNAs that contain an internal lasso [26]. (3) EIciRNAs are nuclear circRNAs that are simultaneously circularized by exons and introns and contain 3′ → 5′ linkage [3] (4) Intergenic circRNAs are another type of non-exon circRNAs found by circRNA identification(CIRI) [24]. However, the mechanism of circRNAs biogenesis still needs more research, revealing potential roles of circRNAs in the crucial processes.

It is known that circRNAs have remarkable characteristics, qualifying circRNAs as valuable biomarkers or targets in the clinics. (1) High stablity: circRNAs exhibit covalently closed loop structures, with absence of 5′-3′ polarity and polyadenylated tails, and develop resistance to RNases, leading to higher stability compared with linear RNAs [21, 27]. (2) Prevalence and specificity: A large variety of circRNAs have been identified to display abundance in various species [28]. The enrichment of circRNAs can also found in saliva and blood [28]. Interestingly, circRNAs are expressed in tissue-specific and developmental stage-specific manners [8, 29]. (3) Conservation: Most circRNAs show high conservation in different species [30].

## Biological roles and mechanistic models of circRNAs

**Fig. 3:**
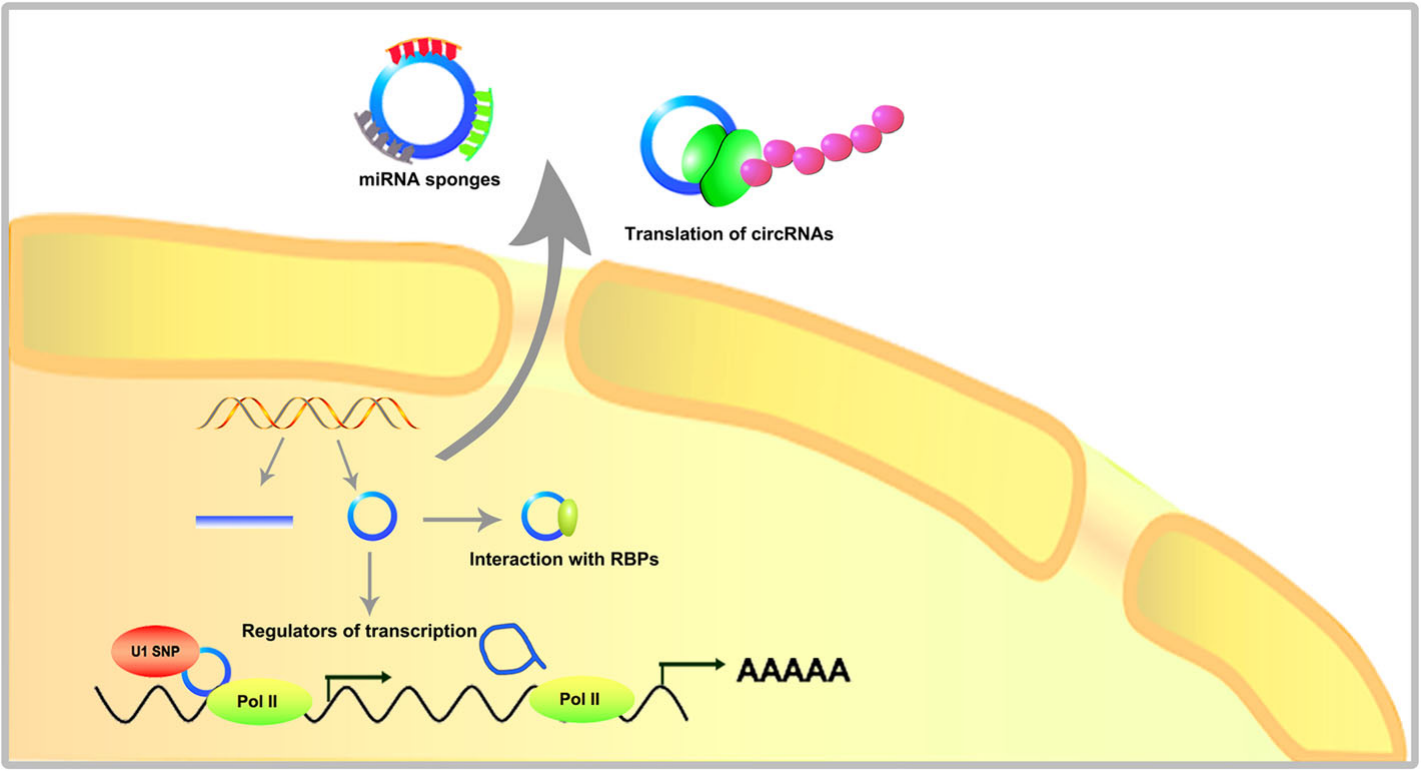
Overview of the the mechanistic models of circRNAs

Emerging studies have identified circRNAs as important regulators in various cancers [31–33]. For instance, For instance, Wang et al. revealed that circRHOT1 inhibited hepatocellular carcinoma (HCC) progression and functioned as a candidate biomarkers for HCC patients [34]. Yi et al. reported that circ-Vimentin (VIM) participates in the regulation of lymphocyte adhesion and transcellular migration in acute myeloid leukemia (AML) [35]. Consistently, receiver operating characteristic (ROC) curve analysis suggested that circ-VIM is an unfavourable prognostic factor for AML patients [35]. Additionally, hsa_circ_0080145 amplification was identified in samples of chronic myeloid leukemia (CML) patients, and the regulatory axis of hsa_circ_0080145/miR-29b may potentially assist in diagnosis and treatments of CML [36]. Indeed, it is widely recognized that circRNAs show great promise as novel biomarkers or targets in multiple cancers [37–39]. Here we comprehensively summarized the dysregulated circRNAs in various tumours in Table 1. Mechanistically, circRNAs can act as miRNA sponges and transcriptional regulators, and interact with RNA-binding protein (RBP) [40–42]. Moreover, a handful of circRNAs can be translated into proteins/peptides [43]. The overview of the mechanistic models of circRNAs is shown in Fig. 3.

**Table 1:** The dysregulated circRNAs in various types of cancers

Symbol	Function	Expression	Cancer Type	Mechanism	PMID
circ-ANAPC7 (hsa_circ_0005785)	/	Upregulated	Acute Myeloid Leukemia.	circ-ANAPC7/miR-181	29,969,755
hsa_circ_0075001	/	Upregulated	Acute Myeloid Leukemia.	/	28,971,903
circ-DLEU2	oncogene	Upregulated	Acute Myeloid Leukemia.	circ-DLEU2/miR-496/PRKACB	30,037,980
hsa_circ_0004277	/	Downregulated	Acute Myeloid Leukemia.	/	28,282,919
circ-HIPK2	/	Downregulated	Acute Myeloid Leukemia.	circ-HIPK2/miR-124-3p	29,844,435
hsa_circ_0075825	/	Upregulated	Basal cell carcinoma	/	27,097,056
hsa_circ_0075828	/	Upregulated	Basal cell carcinoma	/	27,097,056
hsa_circ_0022383	/	Downregulated	Basal cell carcinoma	/	27,298,156
hsa_circ_0022392	/	Downregulated	Basal cell carcinoma	/	27,298,156
circRNA-MYLK(hsa_circ_0002768)	oncogene	Upregulated	Bladder Cancer	circRNA-MYLK/miR-29a/VEGFA/VEGFR2	28,687,357
circTCF25(hsa_circ_0041103)	oncogene	Upregulated	Bladder Cancer	circTCF25/miR-103-3p/miR-107/CDK6	27,484,176
circ-BCRC4	tumor suppressor	Downregulated	Bladder Cancer	circ-BCRC4.miR-101/EZH2	29,270,748
circ-ITCH	tumor suppressor	Downregulated	Bladder Cancer	circ-ITCH/miR-17/miR-224/p21/PTEN	29,386,015
circHIPK3	tumor suppressor	Downregulated	Bladder Cancer	circHIPK3/miR-558/HPSE	28,794,202
circ-ABCB10 (hsa_circ_0008717)	oncogene	Upregulated	Breast Cancer	circ-ABCB10/miR-1271	28,744,405
circ-DENND4C	oncogene	Upregulated	Breast Cancer	HIF1α/circ-DENND4C	28,739,726
circGFRA1 (hsa_circ_0005239)	oncogene	Upregulated	Breast Cancer	circGFRA1/miR-34a/GFRA1	29,037,220
hsa_circ_0001982	oncogene	Upregulated	Breast Cancer	hsa_circ_0001982/miR-143	28,933,584
hsa_circ_0011946	oncogene	Upregulated	Breast Cancer	hsa_circ_0011946/miR-26a/miR-26b / RFC3	29,593,432
circ-Foxo3	tumor suppressor	Downregulated	Breast Cancer	p53/miR-22/ miR-136/ miR-138/ miR-149/ miR-433/ miR-762/ miR-3614-5p/ miR-3622b-5p	27,886,165/26657152
hsa_circ_000911	tumor suppressor	Downregulated	Breast Cancer	hsa_circ_000911/miR-449a/Notch1	29,431,182
hsa_circ_0000284	oncogene	Upregulated	Cervical Cancer	hsa_circ_0000284/miR-506/Snail-2	29,511,454
hsa_circ_0023404	oncogene	Upregulated	Cervical Cancer	hsa_circ_0023404/miR-5047/VEGFA	31,082,770
CDR1as (hsa_circ_0001946,ciRS-7)	oncogene	Upregulated	Cholangiocarcinoma	/	29,424,892
hsa_circ_0001649	tumor suppressor	Downregulated	Cholangiocarcinoma	/	29,337,065
circ-CBFB	oncogene	Upregulated	Chronic Lymphocytic Leukemia	circ-CBFB/miR-607/FZD3/Wnt/β-catenin	29,902,450
circ_0132266	/	Downregulated	Chronic Lymphocytic Leukemia	circ_0132266/miR-337-3p/PML	31,152,142
circ-BA9.3	oncogene	Upregulated	Chronic Myelogenous Leukaemia	circ-BA9.3/ABL1/BCR-ABL1	30,224,298
hsa_circ_0080145	oncogene	Upregulated	Chronic Myelogenous Leukaemia	hsa_circ_0080145/miR-29b	30,205,959
hsa_circ_0001793	/	Upregulated	Colorectal Cancer	/	25,624,062
circ_001569	oncogene	Upregulated	Colorectal Cancer	circ_001569/miR-145/E2F5/BAG4/FMNL2	27,058,418
circ-BANP	oncogene	Upregulated	Colorectal Cancer	circ-BANP/p-Akt	28,103,507
circCCDC66	oncogene	Upregulated	Colorectal Cancer	circCCDC66/miR-33b/ miR-93	28,249,903
circHIPK3	oncogene	Upregulated	Colorectal Cancer	circ-HIPK/miR-7/FAK//IGF1R/ EGFR/YY1	29,549,306
hsa_circ_0000069	oncogene	Upregulated	Colorectal Cancer	/	28,003,761
CDR1as (hsa_circ_0001946, ciRS-7)	oncogene	Upregulated	Colorectal Cancer	hsa_circ_0001946/miR-7/EGFR/ RAF1	25,624,062/28174233
hsa_circ_0007534	oncogene	Upregulated	Colorectal Cancer	/	29,364,478
hsa_circ_000984	oncogene	Upregulated	Colorectal Cancer	hsa_circ_000984/miR-106b/CDK6	29,207,676
hsa_circ_0020397	oncogene	Upregulated	Colorectal Cancer	hsa_circ_0020397/miR-138/TERT/ PD-L1	28,707,774
hsa_circ_0136666	oncogene	Upregulated	Colorectal Cancer	hsa_circ_0136666/miR-136/SH2B1	30,370,521
hsa_circ_0000523	/	Downregulated	Colorectal Cancer	/	25,624,062
hsa_circ_0001346	/	Downregulated	Colorectal Cancer	/	25,624,062
hsa_circ_0001649	/	Downregulated	Colorectal Cancer	/	29,421,663
cir-ITCH	tumor suppressor	Downregulated	Colorectal Cancer	cir-ITCH/miR-7/ miR-20a/ miR-214/Wnt/β-catenin	26,110,611
hsa_circ_0003906	tumor suppressor	Downregulated	Colorectal Cancer	/	29,123,417
cric-FBXW7 (hsa_circ_001988)	tumor suppressor	Downregulated	Colorectal Cancer	/	26,884,878
hsa_circ_0070933	/	Upregulated	Cutaneous squamous cell carcinoma	/	27,298,156
hsa_circ_0070934	/	Upregulated	Cutaneous squamous cell carcinoma	/	27,298,156
hsa_circ_0022383	/	Downregulated	Cutaneous squamous cell carcinoma	/	27,298,156
hsa_circ_0022392	/	Downregulated	Cutaneous squamous cell carcinoma	/	27,298,156
circ-HIPK3	oncogene	Upregulated	Epithelial Ovarian Cancer	/	29,949,144
circ-SLC7A5	oncogene	Upregulated	Esophageal squamous cell carcinoma	/	31,726,270
circPRKCI (hsa_circ_0067934)	oncogene	Upregulated	Esophageal squamous cell carcinoma	/	27,752,108
hsa_circ_0000518	/	Downregulated	Esophageal squamous cell carcinoma	hsa_circ_0000518/miR-181a-2/ miR-512-5p/ miR-521/ miR-556-5p/ miR-663b/ miR-1204	27,465,405
hsa_circ_0000554	/	Downregulated	Esophageal squamous cell carcinoma	hsa_circ_0000554/miR-30c-1/ miR-30c-2/ miR-122/ miR-139-3p/ miR-339-5p/ miR-1912	27,465,405
circ-ITCH	tumor suppressor	Downregulated	Esophageal squamous cell carcinoma	miR-7/ miR-17/ miR-214/Wnt/β-catenin	25,749,389
circPVT1 (hsa_circ_0001821)	/	Upregulated	Gastric Cancer	/	31,616,472
circLMTK2	oncogene	Upregulated	Gastric Cancer	circLMTK2/miR-150-5p/c-Myc	31,722,712
has-circRNA7690–15	oncogene	Upregulated	Gastric Cancer	/	28,980,874
hsa_circ_0047905	oncogene	Upregulated	Gastric Cancer	/	28,980,874
hsa_circ_0138960	oncogene	Upregulated	Gastric Cancer	/	28,980,874
hsa_circ_0001017	/	Downregulated	Gastric Cancer	/	29,098,316
hsa_circ_0001649	/	Downregulated	Gastric Cancer	/	28,167,847
hsa_circ_0003159	/	Downregulated	Gastric Cancer	/	28,618,205
hsa_circ_0014717	/	Downregulated	Gastric Cancer	/	28,544,609
hsa_circ_0061276	/	Downregulated	Gastric Cancer	/	29,098,316
hsa_circ_0074362	/	Downregulated	Gastric Cancer	/	29,240,459
hsa_circ_002059	tumor suppressor	Downregulated	Gastric Cancer	/	25,689,795
circ-LPHN2 (hsa_ circRNA_100269)	tumor suppressor	Downregulated	Gastric Cancer	hsa_ circRNA_100269/miR-630	28,657,541
circ-HuR (hsa_circ_0049027)	tumor suppressor	Downregulated	Gastric Cancer	circ-HuR/CNBP	31,718,709
circLARP4	tumor suppressor	Downregulated	Gastric Cancer	circLARP4/miR-424/LATS1	28,893,265
hsa_circ_0000096	tumor suppressor	Downregulated	Gastric Cancer	hsa_circ_0000096/cyclin D1/CDK6/ MMP2/ MMP9	28,081,541
hsa_circ_0000181	tumor suppressor	Downregulated	Gastric Cancer	/	28,940,688
hsa_circ_0000190	tumor suppressor	Downregulated	Gastric Cancer	/	28,130,019
hsa_circ_0000520	tumor suppressor	Downregulated	Gastric Cancer	/	29,103,021
hsa_circ_0000745	tumor suppressor	Downregulated	Gastric Cancer	/	28,974,900
hsa_circ_0001895	tumor suppressor	Downregulated	Gastric Cancer	/	28,443,463
hsa_circ_0067582	tumor suppressor	Downregulated	Gastric Cancer	/	31,721,300
circ-VCAN	/	Upregulated	Gliomas	/	26,873,924
circ_0001730	oncogene	Upregulated	Gliomas	circ_0001730/miR-326/Wnt7B	31,304,776
circ-SHKBP1	oncogene	Upregulated	Gliomas	circ-SHKBP1/miR-544a/miR-379/FOXP1/FOXP2	29,499,945
circ-ZNF292	oncogene	Upregulated	Gliomas	circ-ZNF292/Wnt/β-catenin	27,613,831
circTTBK2 (hsa_circ_0000594)	oncogene	Upregulated	Gliomas	circTTBK2/miR-217/HNF1β/Derlin-1	28,219,405
cric-FBXW7 (hsa_circ_001988)	oncogene	Upregulated	Gliomas	/	28,903,484
hsa_circ_0046701	oncogene	Upregulated	Gliomas	hsa_circ_0046701/miR-142/ITGB8	29,337,055
CDR1as (hsa_circ_0001946,ciRS-7)	/	Downregulated	Gliomas	miR-671-5p/CDR1as/CDR1/VSNL1	26,683,098
circ-BRAF	/	Downregulated	Gliomas	/	28,236,760
circ-SHPRH	tumor suppressor	Downregulated	Gliomas	/	29,343,848
circSMARCA5(hsa_circ_0001445)	tumor suppressor	Downregulated	Gliomas	circSMARCA5/SRSF1	29,415,469
circRNA_100338	/	Upregulated	Hepatocellular Carcinoma	circRNA_100338/miR-141-3p	31,157,168/28710406
hsa_circ_0000284	/	Upregulated	Hepatocellular Carcinoma	hsa_circ_0000284/miR-124/miR-152 /miR-193a/miR-29a/miR-29b/miR-338/miR-379/miR-584/miR-654	27,050,392
circ_000839	oncogene	Upregulated	Hepatocellular Carcinoma	circ_000839/miR-200b	28,695,771
circPRKCI (hsa_circ_0067934)	oncogene	Upregulated	Hepatocellular Carcinoma	has_circ_0067934/miR-1324/FZD5/Wnt/β-catenin	29,458,020
hsa_circ_0005075	oncogene	Upregulated	Hepatocellular Carcinoma	/	27,258,521
CDR1as (hsa_circ_0001946, ciRS-7)	/	Downregulated	Hepatocellular Carcinoma	hsa_circ_0001946/miR-7/EGFR/CCNE1/PIK3CD	28,892,615/27391479
hsa_circ_0001649	/	Downregulated	Hepatocellular Carcinoma	/	26,600,397
hsa_circ_0004018	/	Downregulated	Hepatocellular Carcinoma	/	28,938,566
hsa_circ_0067531	/	Downregulated	Hepatocellular Carcinoma	/	29,251,325
circC3P1	tumor suppressor	Downregulated	Hepatocellular Carcinoma	circC3P1/miR-4641/PCK1	29,608,893
circMTO1 (hsa_circ_0007874)	tumor suppressor	Downregulated	Hepatocellular Carcinoma	circMTO1/miR-9/p21	28,520,103
circSMARCA5 (hsa_circ_0001445)	tumor suppressor	Downregulated	Hepatocellular Carcinoma	circSMARCA5 /miR-17-3p/miR-181-5p/TIMP3	29,378,234
circZKSCAN1 (hsa_circ_0001727)	tumor suppressor	Downregulated	Hepatocellular Carcinoma	/	28,211,215
hsa_circ_0003570	tumor suppressor	Downregulated	Hepatocellular Carcinoma	/	28,493,512
hsa_circ_0005986	tumor suppressor	Downregulated	Hepatocellular Carcinoma	hsa_circ_0005986/miR-129/Notch1	28,410,211
circRNA_100876	/	Upregulated	Lung Cancer	/	28,343,871
circ-CER	oncogene	Upregulated	Lung Cancer	circ-CER/miR-136/MMP13	28,343,871
circMAN2B2	oncogene	Upregulated	Lung Cancer	circMAN2B2/miR-1275/FOXK1	29,550,475
circPRKCI (hsa_circ_0067934)	oncogene	Upregulated	Lung Cancer	circPRKCI/miR-545/ miR-589/E2F7	29,588,350
circRNA_102231	oncogene	Upregulated	Lung Cancer	/	29,602,132
hsa_circ_0000064	oncogene	Upregulated	Lung Cancer	hsa_circ_0000064/MMP2/MMP9	29,223,555
hsa_circ_0007385	oncogene	Upregulated	Lung Cancer	hsa_circ_0007385/miR-181	29,372,377
hsa_circ_0012673	oncogene	Upregulated	Lung Cancer	hsa_circ_0012673/miR-22/ErbB3	29,366,790
hsa_circ_0013958	oncogene	Upregulated	Lung Cancer	hsa_circ_0013958/miR-134/cyclin D1	28,685,964
hsa_circ_0014130	oncogene	Upregulated	Lung Cancer	/	29,440,731
circRNA-FOXO3	tumor suppressor	Downregulated	Lung Cancer	/	29,620,202
hsa_circ_0013958	tumor suppressor	Downregulated	Lung Cancer	hsa_circ_0013958/miR-7/miR-214/Wnt/b-catenin	27,642,589
circDOCK1	oncogene	Upregulated	Oral Squamous Cell Carcinoma	circDOCK1/mi-196a/BIRC3	29,286,141
hsa_circRNA_103801	/	Upregulated	Osteosarcoma	hsa_circRNA_103801/miR-370	28,957,794
circ_0102049	oncogene	Upregulated	Osteosarcoma	circ_0102049/miR-1304-5p/MDM2	31,727,503
circ-UBAP2	oncogene	Upregulated	Osteosarcoma	circ-UBAP2 /miR-143	28,977,896
circPVT1 (hsa_circ_0001821)	oncogene	Upregulated	Osteosarcoma	circPVT1/ABCB1	29,559,849
hsa_circ_0001564	oncogene	Upregulated	Osteosarcoma	hsa_circ_0001564/miR-29c	29,229,385
hsa_circ_0009910	oncogene	Upregulated	Osteosarcoma	circ-UBAP2/miR-449a/IL-6R/JAK1/STAT3	29,117,539
hsa_circ_0016347	oncogene	Upregulated	Osteosarcoma	hsa_circ_0016347/miR-124/caspase-1	28,424,426
hsa_circRNA_104980	/	Downregulated	Osteosarcoma	/	28,957,794
hsa_circ_0005397	/	Upregulated	Pancreatic cancer	hsa_circ_0005397/miR-26b/miR-125a/miR-181a/miR-330/miR-382	27,997,903
circ-ASH2L	oncogene	Upregulated	Pancreatic cancer	circ-ASH2L/miR-34a/Notch1	31,718,694
circ-LDLRAD3	oncogene	Upregulated	Pancreatic cancer	/	29,307,994
circRNA_100782	oncogene	Upregulated	Pancreatic cancer	circRNA_100782/miR-124/IL6/STAT	29,255,366
circ-ANAPC7 (hsa_circ_0005785)	/	Downregulated	Pancreatic cancer	hsa_circ_0005785/miR-181a/miR-181b/miR-181d/miR-338/miR-526b	27,997,903
circ-SMARCA5	oncogene	Upregulated	Prostate cancer	/	28,765,045
circMTO1 (hsa_circ_0007874)	tumor suppressor	Downregulated	Prostate cancer	circMTO1/miR-17-5p	31,713,278

### miRNA sponges

MiRNAs, a class of abundant short (~ 22 nucleotides) ncRNAs, modulate gene expression by directly base pairing to target sites in mRNAs [44]. In addition to mRNAs, pseudogenes and long non-coding RNAs (lncRNAs), many circRNAs have now been shown to regulate miRNA networks as competitive endogenous RNA (ceRNA) [32, 45, 46]. There are two characteristic circRNAs, namely circular RNA sponge for miR-7 (ciRS-7) and circRNA sex-determining region Y (cir-SRY) [47, 48].

The first circRNA shown to serve as a miRNA sponge was ciRS-7, which contains more than 70 conservative binding sites for miRNA-7 [48]. Recently, it has been observed that ciRS-7 is highly expressed in multiple cancers, including colorectal cancer (CRC), GC, esophageal squamous cell carcinoma (ESCC) and non-small cell lung cancer (NSCLC) [47]. Amplification of ciRS-7 can block the tumour-suppressive activities of miR-7 and antagonize miR-7-meddiated phosphatase and tensin homolog (PTEN)/phosphatidylinositol 3-kinase (PI3K)/AKT pathway, resulting in a more aggressive oncogenic phenotype in GC [49]. Additionally, Sang et al. revealed that ciRS-7 amplification drives ESCC progression partly through targeting miR-876-5p/MAGE-A family axis [50]. Another study concerning the role of ciRS-7 in ESCC demonstrated that ciRS-7 can prompt ESCC progression through miR-7/HOXB13/NF-κB or miR-7/KLF4/NF-κB axis, providing novel prognostic indicators and therapeutic targets for ESCC patients [51, 52]. As for cir-SRY, a looped RNA specifically expressed in the mouse testis, it includes sixteen binding sites of miR-138. The formation of cir-SRY/miR-138 axis was shown to be a general phenomenon [48].

Currently, a large number of circRNAs have been confirmed to serve as miRNA sponges, proposing a possible view that such a mechanism may be common to all circRNAs [53–55]. Shortly thereafter, following studies refuted the previously hypothesis, asserting that most circRNAs are not able to serve as miRNA sponges [23, 56, 57]. The main reason accounting for this assertion is that only a small portion of circRNAs possess multiple binding sites for specific miRNAs [23, 56, 57]. Gained from the most recently knowledge, we conclude that some circRNAs can behave as a sponge of specific miRNAs and induce the suppression of miRNAs. There are also other mechanisms for the regulation of circRNAs, since the mechanism of circRNAs as miRNA sponges is not existed in isolation.

### Interaction with RBPs

In addition to miRNA “sponge” property, circRNAs can also bind to RNA-binding proteins (RBPs), competitively blocking protein-active elements in a sequence-specific manner [16, 58]. Current investigations have illuminated that circRNA polyadenylatebinding nuclear protein 1 (circ-PABPN1) can bind to human antigen R (HUR) [41]. This extensive interaction displayed suppressive effects on the binding of HuR to PABPN1 mRNA, thus partially or completely blocking the translation of PABPN1 [41]. Another study showed that circRNA forkhead box O3 (circ-Foxo3) functioned as a scaffold and formed a circ-Foxo3-P21-cyclin dependent kinase 2 (CDK2) ternary complex, thus avoiding the formation of cyclin E/CDK2 complex [58]. This interacted complex can impact on cell proliferation and cell cycle regulation [58]. In addition, most recent studies also highlight that circ-Amotl1 can determine the subcellular translocation of several RBPs, such as pyruvate dehydrogenase kinase 1 (PDK1), MYC, and signal transducer and activator of transcription 3 (STAT3) [59–61].

### Regulators of transcription

Many scholars have discovered that circRNAs can also regulate gene expression at transcriptional or post-transcriptional level [42, 62]. EIciRNAs and ciRNAs, which are primarily located in the nucleus, are likely to function at the transcriptional level [25, 26, 42]. For instance, EIciRNAs, such as circRNA eukaryotic translation initiation factor 3 subunit J (circ-EIF3J) and circRNA poly(A) binding protein interacting protein 2 (circ-PAIP2), are demonstrated to interact with RNA Pol II in combination with U1 snRNP to enhance the expression of their parental genes [42, 63]. Similarly, this mechanistic model can be found in ciRNAs and their parental gene. CiRNAs, such as ci-ankrd52 and ci-sirt7, interacted with Pol II and positively mediated the transcription of parental genes [26]. Of note, the regulation of transcription may be a common mechanism for circRNAs.

### Translation of circRNAs

CircRNAs were initially recognized as ncRNAs without protein-coding abilities, due to the absence of a 5′ cap structure and poly(A) tail [43]. Interestingly, researchers uncovered the translation capacity of some circRNAs, which may carry open reading frame (ORF) [43]. Further studies found that circRNA zinc finger protein (circ-ZNF609), a functional circRNA expressed in mouse and human myoblasts, can translate proteins in mouse myoblasts driven by an internal ribosome entry site (IRES) [64]. This detection supplies the first determination that endogenous circRNAs exhibit protein-encoding abilities [64]. Later, the circRNA F-box and WD repeat domain containing 7 (circ-FBXW7) was found to be translated into the new 21 kDa protein FBXW7 [65]. These discoveries indicate new capabilities for circRNAs and provide a new direction for the future of circRNA research.

## CircRNAs as modulators of the tumour microenvironment

**Fig. 4:**
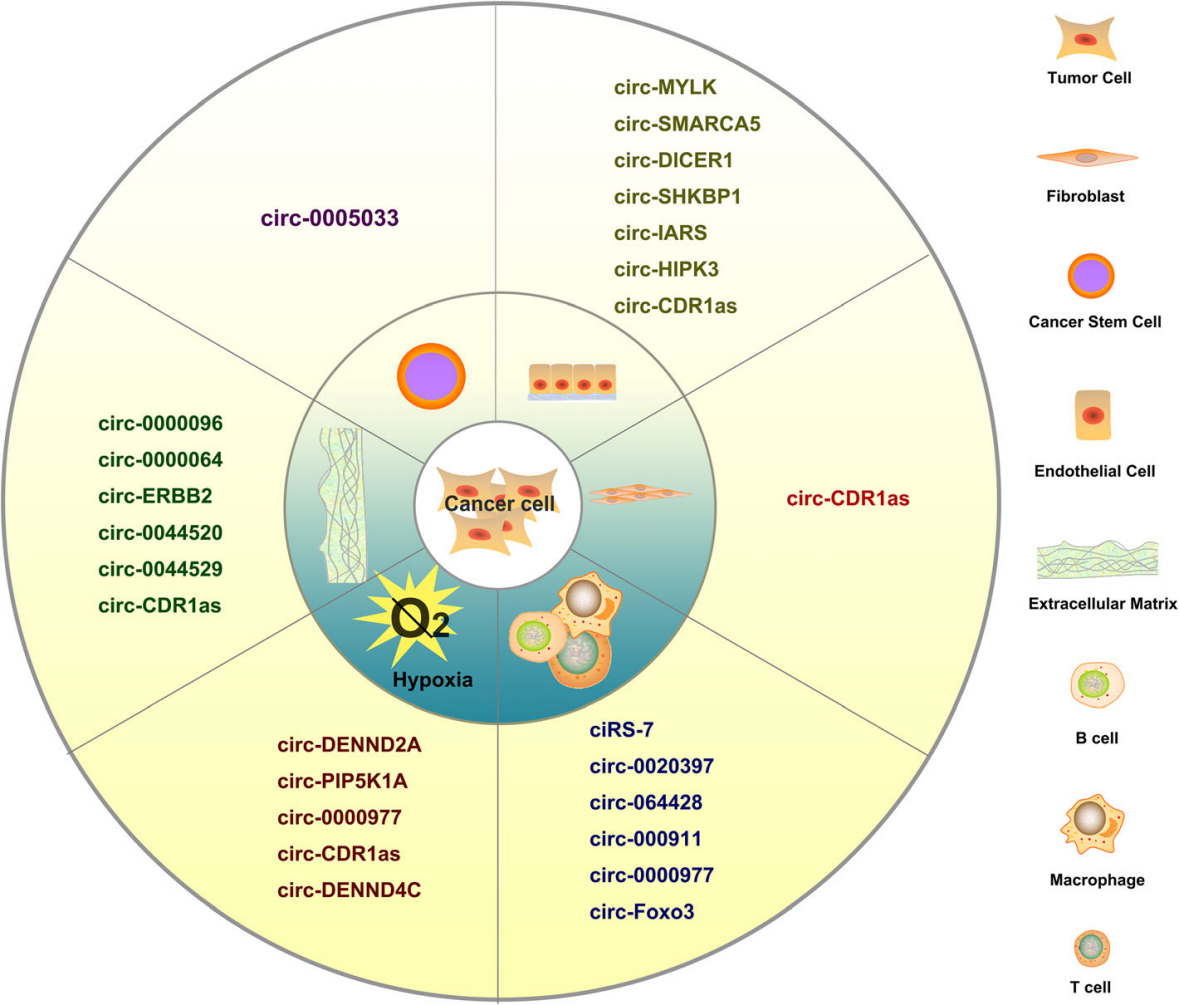
The communication mediated by circRNAs between tumour cells and the TME

TME includes different cellular and non-cellular secreted components [12, 66]. The cellular components of TME include CAEs, immune cells, CAFs, and CSCs [12, 66]. Secreted non cellular components comprise cytokines, growth factors, metabolites and ECM proteins [12, 66]. There is an increasing mount of evidence showing the complex interaction between circRNAs and key counterparts in the TME. The importance and ubiquity of this interaction is just beginning to be realized, and warrant further investigation to develop new targets and cancer therapeutics. Here we are first systematically reviewing the interplay between circRNAs and cellular/non-cellular counterparts of the TME. The communication mediated by circRNAs between tumour cells and the TME is shown in Fig. 4. The emerging role and mechanistic axis of circRNAs associated with the TME is listed in Table 2.

**Table 2:** The emerging role and mechanistic axis of circRNAs associated with the TME

Interaction with TME	CircRNA name	Expression	Function	Cancer types	Biological activities	Clinical correlation	Molecular axis
circRNAs and CAEs	circ-MYLK	Upregulated	oncogene	Breast cancer	Proliferation, migration, apoptosis, angiogenesis, metastasis, EMT	TNM stage, pathological grade	circ-MYLK/miR-29a/VEGFA/VEGFR2
	circ-SMARCA5	Downregulated	tumor suppressor	Glioblastoma multiform	Angiogenesis	Overall Survival, Progression-Free Survival	circ-SMARCA5/SRSF1/VEGFA
	circ-DICER1	Upregulated	oncogene	Glioma	Proliferation, migration, angiogenesis	/	MOV10/circ-DICER1/ miR-103a-3p/miR-382-5p/ZIC4
	circ-SHKBP1	Upregulated	oncogene	Glioma	Proliferation, migration, angiogenesis	/	circ-SHKBP1/miR-544a/FOXP1, circ-SHKBP1/miR-379/FOXP2
	circ-IARS	Upregulated	oncogene	Pancreatic ductal adenocarcinoma	Invasion, metastasis, endothelial monolayer permeability	Vascular invasion, TNM stage, liver metastasis, postoperative survival time	circ-IARS/miR-122/RhoA/F-actin/ZO-1
	circ-HIPK3	Downregulated	tumor suppressor	Bladder cancer	Proliferation, migration, invasion, angiogenesis, metastasis	Pathological grade, tumor invasion, lymph node metastasis	circ-HIPK3/miR-558/HPSE
	ciRS-7	Upregulated	oncogene	Multiple tumours	Angiogenesis, immune and stromal infiltration, ECM organization, integrin and collagen binding	/	cir-DR1as/TGF-β signaling pathway/ECM–receptor interaction
circRNAs and immune cells	ciRS-7	Upregulated	oncogened	Esophageal squamous cell carcinoma	Proliferation, migration, invasion, metastasis	TNM stage, pathological grade, overall survival, disease-free survival	ciRS-7miR-7/KLF4/NF-κB
				Multiple tumours	Angiogenesis, immune and stromal infiltration, ECM organization, integrin and collagen binding	/	cir-DR1as/TGF-β signaling pathway/ECM– receptor interaction
	has_circ_0020397	Upregulated	oncogene	Colorectal cancer	Proliferation, invasion, apoptosis	/	has_circ_0020397/miR-138/TERT/PD-L1
	hsa_circ_0064428	Downregulated	tumor suppressor	Hepatocellular carcinoma	/	Tumour size, metastasis, overall survival	/
	circRNA-000911	Downregulated	tumor suppressor	Breast cancer	Proliferation, migration and invasion, apoptosis	/	circ-000911/ miR-449a /Notch1/NF-κB
	circ-0000977	Upregulated	oncogene	Pancreatic cancer	Immune escape, induction by hypoxia	/	circ-0000977/miR-153/HIF1α/ADAM1
	circ-Foxo3	Downregulated	tumor suppressor	Breast cancer	Proliferation, apoptosis, tumor growth	/	circ-Foxo3/MDM2/P53/Foxo3
CircRNAs and CAFs	ciRS-7	Upregulated	oncogene	Multiple tumours	Angiogenesis, immune and stromal infiltration, ECM organization, integrin and collagen binding	/	cir-DR1as/TGF-β signaling pathway/ECM– receptor interaction
circRNAs and CSCs	hg19_circ_0005033	Upregulated	oncogene	Laryngeal squamous cell carcinoma	Proliferation, migration, invasion, chemotherapy resistance	/	hg19_circ_0005033/miR-4521/miR-339-5p/STA T5A
circRNAs and hypoxia	circ-DENND2A	Upregulated	oncogene	Glioma	Migration, invasion, induction by hypoxia	/	circ-DENND2A/miR-625-5p/HIF1α
	circ-PIP5K1A	Upregulated	oncogene	Non-small cell lung cancer	Proliferation, invasion, migration, metastasis, EMT	/	circ-PIP5K1A/miR-600/HIF-1α
	circ-0000977	Upregulated	oncogene	Pancreatic cancer	Immune escape, induction by hypoxia	/	circ-0000977/miR-153/HIF1α/ADAM1
	circ-CDR1as	Downregulated	tumor suppressor	Ovarian cancer	Proliferation, migration, invasion	/	circ-CDR1as/miR-135b-5p/HIF1AN
	circ-DENND4C	Upregulated	oncogene	Breast cancer	Proliferation, induction by hypoxia	/	HIF1α/circ-DENN4C
circRNAs and ECM	hsa_circ_0000096	Downregulated	tumor suppressor	Gastric cacner	Proliferation, migration	Gender, invasion, TNM stage	hsa_circ_0000096/MMP2/MMP9
	hsa_circ_0000064	Upregulated	oncogene	Non-small cell lung cancer	Proliferation, cell cycle, apoptosis, migration, invasion	T stage, TNM stage, lymph node metastasis	hsa_circ_0000064/MMP2/MMP9
	ciRS-7	Upregulated	oncogene	Multiple tumours	Angiogenesis, immune and stromal infiltration, ECM organization, integrin and collagen binding	/	cir-DR1as/TGF-β signaling pathway/ECM– receptor interaction
	circ-ERBB2	Upregulated	oncogene	Gastric cacner	Proliferation, apoptosis, migration, invasion	Tumor size, invasion depth, overall survival	circ-ERBB2/miR-637/MMP-19
	hsa_circ_0044520	Upregulated	/	Laryngeal Squamous Cell Carcinoma	Collagen synthesis	/	hsa_circ_0044520/COL1A1
	hsa_circ_0044529	Upregulated	/	Laryngeal Squamous Cell Carcinoma	Collagen synthesis	/	hsa_circ_0044529/COL1A1

### CircRNAs and CAEs

CAEs are an important component of the tumour stroma in the TME [12]. CAEs are arranged on the inner surface of tumour blood vessels and lymphatic vessels, and can be responsible for supporting blood vessel formation and tumour neovasculature [67]. It is widely believed that angiogenesis is an important mechanism by which tumours not only remove carbon dioxide and metabolic waste but also obtain adequate nutritional support [68]. Overexpression of various angiogenic factors and the rapid growth of tumour cells in the TME can lead to the development of vascular networks with many structural and functional abnormalities [69, 70]. Vascular endothelial growth factor (VEGF), which is amplified in various cancers, has been recognized to be essential for both physiological and pathological angiogenesis [71, 72]. Moreover, targeting of inhibitors associated with PI3K/Akt signaling pathway can dramatically reduce the secretion of VEGF, leading to suppressive effects on angiogenesis [73]. CircRNAs involved in the TME can affect diverse physiological and pathological activities, including tumour angiogenesis [17, 74].

Most recently, Zou et al. identified the effects of circ-CDR1as on angiogenesis, with a positive correlation between circ-CDR1as and infiltrating level of CAEs [75]. Another research by Zhong et al. showed that circRNA myosin light chain kinase (circ-MYLK) could sponge miR-29a to relieve suppression for targeting VEGFA in breast cancer, thus promoting tumour angiogenesis [17]. This circ-MYLK-mediated ceRNA network would provide promising target for BC diagnosis and therapy [17]. Additionally, current evidence shows that circRNA SMARCA5 (circ-SMARCA5) can bind to serine and arginine rich splicing factor 1 (SRSF1) to regulate VEGFA pathway in glioblastoma multiforme (GBM) cells [16]. Specifically, SRSF1 can identify the proximal splice site (PSS) of VEGF, and elevate the expression of pro-angiogenic isoforms (VEGF-Axxxa), thus contributing to tumour angiogenesis [16].

In addItion to VEGF, other angiogenic factors may also affect CAEs directly or indirectly [76–81]. Recent studies reveal that glioma-exposed endothelial cells (GECs) exhibit high expression of circRNA DICER1 (circ-DICER1) and its RBP MOV10 [82]. Of note, circ-DICER1 could sponge miR-103a-3p/miR-382-5p and attenuated the negative regulation of Zic family member 4 (ZIC4), thus mediating cell proliferation, migration and angiogenesis of GECs [82]. This molecular axis of MOV10/circ-DICER1/miR-103a-3p/miR-382-5p/ZIC4 gives novel insights into glioma angiogenesis, providing prospective targets for anti-angiogenesis strategy [82]. Another circRNA axises linked to glioma tumourigenesis and angiogenesis are the circ-SHKBP1/miR-544a/FOXP1 and circ-SHKBP1/miR-379/FOXP2 pathway [83]. It was shown that U87 GECs displayed high circ-SHKBP1 expression, and circ-SHKBP1 knockdown exhibited inhibitory effects on malignant phenotype and tube-formation capacities of GECs [83]. Specifically, FOXP1/FOXP2 can elevate the expression of angiogenic factor angio genic factor with G patch and FHA domains 1 (AGGF1), which can promote GEC activity, tube formation and migration through PI3K/AKT and extracellular signal-regulated kinase 1/2 (ERK1/2) pathways [83]. It can be concluded that circ-SHKBP1-mediated regulatory axis may offer potential targets and molecular-based therapy for combined treatment of glioma [83].

Emerging studies have elucidated that aberrant expression of circRNAs can play oncogenic or anti-tumour functions in various cancers [31, 84]. Li et al. identified circRNA homeodomain interacting protein kinase 3 (circ-HIPK3) to be significantly decreased in 79.5% of the bladder cancer tissues and cell lines, revealing its underlying tumour-suppressive roles [85]. Interestingly, low circ-HIPK3 expression was positively associated with tumour grade, invasion, and the lymph node metastasis, suggesting its potential clinical value as a novel biomarker for early diagnosis and targeted therapy [85]. Mechanistically, circ-HIPK3 enrichment can abundantly sponge miR-558 to decrease heparanase (HPSE), VEGF, and matrix metallopeptidase 9 (MMP9) levels, thus effectively suppressing the invasive abilities and angiogenesis of bladder cancer cells [85]. Moreover, circRNA isoleucine-tRNA synthetase (circ-IARS) was detected to be increased in tissues and plasma exosomes of patients with pancreatic cancer (PC) [86]. PC cell-derived exosomal circ-IARS could be delivered to endothelial cell, enhancing vascular invasion [86]. Moreover, circ-IARS can also destory tight junctions between the endothelium cells, leading to increased permeability of vascular endothelial cells and tumour metastasis promotion [86]. Endothelial cells are critical for tumour angiogenesis, and the effects of circRNAs on endothelial cells can affect tumour progression. These findings remind us that targeting circRNAs in the CAEs may be a new approach to cancer therapy.

### CircRNAs and immune cells

Immune cells, which represent the most abundant cellular component of the TME, have been a target of interest for their potent cytotoxic capabilities [87–89]. The crosstalk among cancer cells, immune cells as well as released factors may be involved in the regulation of tumour immunity, and establish an environment that facilitate cancer development and progression [90, 91]. Identifying key regulators within this crosstalk may provide prime candidates for therapeutic intervention [92, 93]. Recently, a variety of circRNAs associated with the TME are determined to be significantly dysregulated in various cancers [17, 83]. Moreover, some circRNAs have been found to interact with immune cells, providing the evidence for the role of circRNAs in the regulation of immune cells [94, 95].

#### CircRNAs and macrophages

It is known that mammalian macrophages can be mediated to various phenotypes under different external stimuli [96]. Current discovery revealed by Zhang et al. has provided the first evidence for the expression pattern of circRNAs in macrophage activation [97]. The expression pattern of circRNAs was explored in response to stimuli polarizing two distinct patterns of macrophage activation (M1 and M2) through circRNA microarray [97]. The validation assay indicated that high levels of circRNA-003780, circRNA-010056 and circRNA-010231 were detected in M1 cells [97]. Similarly, the expression levels of circRNA-003424, circRNA-013630, circRNA-001489 and circRNA-018127 were also detected in M2 cells with fold-change > 5 [97]. Differences in the expression of circRNAs in macrophages of different polarization states were confirmed, providing novel insight into the role of circRNAs in macrophage differentiation and polarization [97]. In addition, another study reported by Zou et al. showed that high level of circ-CDR1as predicted a higher ratio M2 macrophage, suggesting the oncogenic mechanism of circ-CDR1as in regulating the TME [75].

#### CircRNAs and lymphocytes

Multiple reports have elucidated that tumour infiltrating lymphocytes (TILs) display high proportion in the TME, contributing to better overall survivals [98, 99]. It was demonstrated that HCC patients with higher percentage of TILs displayed better clinical outcomes, suggesting the prognostic value of TILs for HCC patients [100]. Then, Weng et al. performed global circRNA microarray between plasma of HCC patients with high TILs and low TILs [101]. Through validation assays, low hsa_circ_0064428 expression was found in HCC patients with high TILs and exhibited close correlation with overall survival, tumour size and metastasis in patients with HCC [101]. It can be concluded that hsa_circ_0064428 functioned as a novel immune-associated prognostic biomarker for HCC patients [101].

Programmed death-ligand 1/programmed death-1 (PD-L1/PD-1), as key immune checkpoints, can suppress the activation of T lymphocytes and increase the immune tolerance of tumour cells, thereby achieving tumour immune escape [102–104]. Studies have increasingly shown that some circRNAs can induce PD-L1 expression in the TME and mediate the regulation of tumour immunity. Zhang et al. revealed that hsa_circ_0020397 was able to elevate the expression of PD-L1 and telomerase reverse transcriptase (TERT) by sequestering miR-138 in CRC cells [62]. Specifically, hsa_circ_0020397-mediated upregulation of PD-L1 can lead to the inhibition of apoptosis and acquisition of tumour immune escape in the TME [62]. The regulatory axis of hsa_circ_0020397/miR-138/TERT/PD-L1 can help to further the theory of tumour immune escape and develop attractive strategies for CRC patients [62]. Shortly afterward, Du et al. reported the tumor-suppressive role of circ-Foxo3 in breast carcinoma [105]. According to histological analysis, the infiltration of B- and T cells into the tumours and surrounding connective tissues expressed circ-Foxo3, suggesting an immune response of the hosts to the tumour xenografts [105].Current data provided by Zou et al. showed that circ-CDR1as takes an important role in immune cell infiltration in tumour tissues, especially those of CD8+ T cells [75]. Furthermore, abnormally expressed exosomal circRNAs may induce Treg cells, and directly interact with immune factors to mediate immune activity, achieving cell-to-cell communication [106, 107]. For instance, immune factors NF90/NF110 can activate circRNA production in the nucleus, and associate with mature circRNAs in the cytoplasm [94]. Upon viral infection, the depression of circRNA expression can be partly accounted by the nuclear export of NF90/NF110 to the cytoplasm [94]. The complex of NF90/NF110-circRNP accumulations can be found in the cytoplasm, and circRNAs can compete with viral mRNAs for binding to NF90/NF110, thus affecting host immune response [94]. Based on the studies above, circRNA may serve as a new tumour antigen, which may be induce the regulation of tumour immunity and develop a new cancer therapy.

#### CircRNAs and immune-related molecules

NF-κB is identified to mediate cellular stress responses, cytokines production, and the process of immune response [108, 109]. The unique role of NF-κB has been indicated in tumour cells and immune cells, such as macrophages and dendritic cell (DCs) [109–111]. As we have mentioned, ciRS-7 has been reported to be abundant in ESCC [47]. Apart from this, inhibition of NF-κB can attenuate MMP-2 upregulation induced by ciRS-7, thus suppressing ciRS-7-mediated invasion of ESCC cells [51]. Another study reported by Wang et al. identified that NF-κB signaling is a functional target of circRNA-000911/miR-449a axis, which may be partly responsible for the oncogenic activities in breast cancer cells [20]. To summarize, the network of circRNA-000911/miR-449a/Notch1/NF-κB may allow for a new direction of therapeutic strategy for breast cancer [20].

#### CircRNAs and exosomes

Exosomes specifically refer to discoidal vesicles with a diameter of 30–150 nm, mediating the communication between immune cells and tumour cells [112, 113]. It is interesting to note that the circRNAs can specifically bind to tumour-specific miRNAs or mRNAs in exosomes, which can serve as new tumour antigens for regulating immune response [94]. Current studies show that a variety of circRNAs were obviously decreased in KRAS mutant cells and can be transferred to exosomes secreted from tumour cells [114].

### CircRNAs and Cancer stem cells

CSCs, which are thought to be the origin of cancer cells, are the driving force for tumour growth, migration, metastasis and therapeutic resistance in multiple cancers [115–117]. It was found that CD133+ CD44+ CSCs (named TDP cells) isolated from the laryngeal squamous cell carcinoma (LSCC) cells could exert promotion effects on cell proliferation, migration, as well as resistance to chemotherapy and irradiation [118]. Moreover, stronger malignant behaviors can be observed in TDP cells compared with that in CD133+ or CD44+ LSCC stem cells, CD133-CD44- LSCC cells (named TDN cells), and parental TU-177 cells (named TPT cells) [118]. It is extremely worthwhile to determine the underlying mechanism of LSCC stem cells [118]. Therefore, Wu et al. uncovered that the core node of the circRNA-miRNA-mRNA regulatory network that enriched in these biological processes and pathways might be important contributors of the enhanced malignancy of LSCC stem cells [118]. Specifically, hg19_circ_0005033 was determined to interact with miR-4521/miR-339-5p in LSCC stem cells, leading to upregulation of STAT5A, which can induce stem-like cell properties and the epithelial-to-mesenchymal (EMT) transition in cancer [118, 119].

It can be concluded that the hg19_circ_0005033/miR-4521/miR-339-5p/STAT5A axis supports the malignant features of LSCC stem cells [118]. The investigation of circRNAs in the regulation of CSCs would give new insights into the molecular mechanisms of CSCs, and provide potential biomarkers and targets for cancer patients.

### CircRNAs and CAFs

CAFs are fibroblasts in a state of continuous activation [120]. As one of the most important components of mesenchyme, CAFs play an important role in tumour growth, proliferation and metastasis [120, 121]. In turn, tumour cells can promote fibroblast activation through feedback regulation [122]. Recent studies have revealed that circRNAs can be found in the normal fibroblasts (NFs). Du et al. reported that hsa_circ_0001946, which was obviously upregulated in lung cancer tissues, was also confirmed to be highly-expressed in the human normal lung fibroblasts MRC-5, compared with that of human NSCLC cell line A549 [123]. Further research validated that hsa_circ_0001946 suppressed progression and induced cisplatin sensitivity in A549 cells, which indirectly provide the basis of interaction between hsa_circ_0001946 and CAFs [123]. In addition, Du et al. revealed that the level of circ-Foxo3 was significantly reduced in NIH3T3 and MEF fibroblasts compared with cancer cell lines [58]. Interestingly, NIH3T3 fibroblasts treated with cell proliferating factor Epidermal growth factor (EGF) displayed decreased circ-Foxo3 expression [58]. Correspondingly, the level of circ-Foxo3 exhibited obvious elevation when the cells are treated with EGF inhibitor AG1478 [58].

Mounting studies have revealed that dysregulation of miRNAs and exosomal miRNAs can interact with the CAFs and participate in the regulation of secretory phenotype, tumourigenesis, metastatic progression, drug resistance and poor prognosis [124–126]. And it is commonly known that circRNAs can function as miRNA sponges to function in various cancers [9, 85]. Thus, it can be inferred that the axis of circRNA/miRNA can be involved in the TME through interacting with CAFs. A recent research by Zou et al. identified a positive correlation between circ-CDR1as expression and the infiltrating level of CAFs [75]. It was shown that circ-CDR1as may serve as a miRNA sponge to form a ceRNA network, thus mediating the TME [75]. The breakthrough in this attractive field may pave a new path for cancer patients based on the targeted therapy against CAFs.

### CircRNAs and extracellular matrix

ECM, including collagen, fibronectin, laminin, glycosaminoglycans and proteoglycans, is an important tissue barrier for tumour invasion and metastasis [127, 128]. MMPs are involved in the turnover and remodeling of ECM and serve as key regulators in the pathological destruction of various tumours [129–131]. Hsa_circ_0000096 knockdown was reported to suppress GC cell migration and invasion through decreasing MMP-2 and MMP-9 expression [132]. Moreover, aberrant expression of hsa_circ_0000096 was closely associated with tumour invasion, TNM stage and gender, revealing its potential utility as prospective markers [132]. A similar research showed that high level of hsa_circ_0000064 was positively correlated with T stage, TNM stage, and lymphatic metastasis in NSCLC patients, and promoted NSCLC cell migration and invasion partly through regulating MMP2/MMP9 expression [133]. Furthermore, Li et al. revealed that upregulation of circ-ERBB2 predicts unfavorable prognosis and activates GC progression partly through miR-637/MMP-19 and miR-503/CACUL1 pathway [134].

Currently, some circRNAs are demonstrated to be involved in the ECM regulation. Zou et al. reported that circ-CDR1as participates in the regulation of ECM organization, integrin binding, and collagen binding [75]. Pathway analysis further identified the involvement of circ-CDR1as in ECM-receptor interaction [75]. In addition, Fan et al. identified 21 dysregulated circRNAs associated with collagen formation in human LSCC tissues through analysing circRNA profiling data [135]. Among these host genes, hsa_circ_0044520 and hsa_circ_0044529 are hosted in the collagen type I alpha 1 chain (COL1A1) gene, which can encode the subunit of type I collagen and regulate the tumourigenesis of various cancers [135]. These interactions may further the identification of ECM in the TME and allow for more opportunities for patients [135]. However, the interplay between circRNAs and ECM still requires extensive investigation.

### CircRNAs and hypoxia

Cancer cells and stromal cells in the TME often limit the access to nutrients and oxygen, contributing to a hypoxia environment [136, 137]. Hyperpoic-inducible factor 1α (HIF1α), a hallmark of hypoxia, exerts great influences on cancer pathobiology [138, 139]. Recently, hypoxia-activated circ-DENND2A was revealed to promote the migratory and invasive capacities of glioma cells through competitively binding to miR-625-5p [55]. Su et al. further showed that circ-DENND2A was dramatically increased in glioma tissues with high level of HIF1α [55]. Moreover, another study showed that the circ-0000977 level can be induced by hypoxia in pancreatic cancer cells [140]. The axis of circ-0000977/miR-153 can mediate HIF1α-induced immune escape of pancreatic cancer cells through targeting HIF1α/ADAM10 [140]. Similarly, Chi et al. demonstrated that circ-PIP5K1A/miR-600/HIF1α axis prompted NSCLC proliferation and metastasis and offer potential targets for NSCLC patients [53]. In ovarian cancer, circ-CDR1as was significantly lower in tumour tissues, and functioned as a sponge of miR-135b-5p to increase the expression of hypoxia-inducible factor 1-alpha inhibitor (HIF1AN), thus exerting inhibitory role on proliferation capacity of ovarian cancer cells [141]. In addition, Liang et al. reported that circ-DENND4C, a HIF1α-associated circRNA, was able to promote the proliferation of breast cancer cells under hypoxia [142]. Importantly, high circ-DENND4C level was positively correlated with larger tumour size in breast cancer patients [142]. Understanding the mechanism of circRNAs under hypoxia may provide further evidence for the potential utilities of circRNAs.

## The potential of circRNAs as prospective biomarkers or targets in cancer clinics

Current studies have shown the great potential of circRNAs as novel biomarkers. Unlike linear RNA molecules, circRNAs possess covalently closed loop structures with high stabilities, avoiding degradation induced by exonuclease RNaseR [143]. Moreover, circRNAs can be detected in tissue samples, saliva or plasma with cell-specific or stage-specific expression pattern [144]. These characteristics can partly account for the possible application of circRNAs as prospective biomarkers. Hsiao et al. found that circCCDC66 was obviously increased in CRC tissues and its amplification predicted poor prognosis for patients with CRC [84]. Further analysis of the receiver operating characteristic (ROC) curve demonstrated that circCCDC66 may be diagnostic biomarker for CRC patients [84]. Another study by Wang et al. detected the elevated circSETDB1 expression in serous ovarian cancer (SOC) patients [145]. It is worth noting that high level of serum circSETDB1 can distinguish SOC patients from healthy individuals [145]. The serum circSETDB1 may become a prospective non-invasive biomarker for SOC patients [145]. Interestingly, circRNAs are stably overexpressed in exosomes, such as circ-RASSF2, circ-PTGR1 and circ-IARS [86, 146, 147]. For instance, Li et al. demonstrated the high level of exosomal circ-IARS in pancreatic cancer tissues and plasma exosomes, suggesting the diagnostic value of exosomal circ-IARS as a promising biomarker [86]. To summarize, circRNAs may have a potential to be developed into effective biomarkers.

An increasing number of studies have highlighted circRNAs as oncogenic or tumour-suppressive regulators in multiple cancers [33, 148, 149]. Nowadays, studies have focused on the application of circRNAs as therapeutic targets [10, 62]. The effective techniques of gene knockdown or overexpression may shed new light on the targeting of circRNAs. For oncogenic circRNAs, specific siRNAs or shRNAs targeting the back-splice junction, which can avoid the interference of homologous linear mRNA expression, were used to achieve circRNA-specific knockdown [150]. Additionally, CRISPR-Cas13a, a flexible platform, was applied to implement programmable knockdown with reduced off-target impacts [151]. As for intron circularized circRNAs, complementary paired siRNAs were designed targeting intron region sequences to disrupt RNA formation, leading to circRNA knockdown [152]. For tumour-suppressive circRNAs, the overexpression vectors prompting back-splicing comprised circRNA-forming exons and flanking introns with reverse complementary sequences [3]. The implication of cis strategy can offer an accurate approach to study the target gene [153]. The replacement of the original weak promoter or a weak intronic RNA with a corresponding strong counterpart can facilitate amplification of circRNAs [153]. To summarize, circRNAs show great potential as therapeutic targets and targeting of circRNAs may become a new model for future cancer treatment. However, extensive work should be done to develop advanced techniques and effective drugs targeting circRNAs.

## circRNAs as novel targets reversing drug resistance for cancer therapeutics

Drug resistance is a huge obstacle of the treatment of tumours, and circRNAs are important players in regulating drug resistance [154]. The emerging role and mechanistic axis of circRNAs associated with drug resistance is shown in Table 3. Liu et al. found that circRNA-MTO1 can interact with tumour necrosis factor receptor associated factor 4 (TRAF4) to decrease Eg5 protein, thereby reversing the resistance to monastrol in breast cancer cells [155]. Another research of breast cancer showed that knockdown of circ-0006528 can obviously increase the sensitivity of Adriamycin (ADM)-resistant cell lines to ADM [148]. Through RNA-seq and bioinformatic analysis, Zhu et al. identified 80 significantly altered circRNAs in osteosarcoma (OS), which may closely associated with chemotherapy resistance [156]. It was confirmed that hsa-circ-0001258/hsa-miR-744-3p/glutathione S-transferase mu 2 (GSTM2) axis suppressed the Doxorubicin (DXR) resistance of OS cells [156]. In addition, circ-PVT1 knockdown was found to weaken the resistance to doxorubicin and cisplatin of OS cells through decreasing the expression of classical drug resistance-related gene ABCB1 [157]. As for the chemoradiation resistance in CRC, Xiong et al. investigated circRNA profiles in CRC cells with resistance to 5-fluorouracil- (5-FU) [158]. Microarray analysis showed that 47 circRNAs were significantly increased and 24 circRNAs were dereased in 5-FU resistant CRC cells, with fold change > 2 [158]. Intriguingly, Xiong et al. also speculated the regulatory axis of hsa_circ_0000504/hsa-miR-485-5p/STAT3 in CRC and downregulation of hsa_circ_0000504 would be a possible option to overcome 5-FU resistance in CRC [158]. The establishment of this database may be useful to discover effective targets to overcome drug resistance of CRC cells [158]. Recently, Shang et al. found a detailed circPAN3/miR-153-5p/miR-183-5p/X-linked inhibitor of apoptosis protein (XIAP) interaction in AML, which can be used as a novel target for reversing ADM resistance in AML patients [159]. However, extensive studies are urgently needed to further the understanding of circRNAs-associated drug resistance in various cancers.

**Table 3:** The emerging role and mechanistic axis of circRNAs associated with drug resistance in various tumours

CircRNA name	Expression	Function	Cancer types	Biological activities	Clinical correlation	Molecular axis
circ-PVT1	Upregulated	oncogene	Osteosarcoma	Resistance to doxorubicin and cisplatin	Enneking stage, lung metastasis, overall survival	circ-PVT1/ABCB1
hsa_circ_0004015	Upregulated	oncogene	Non-small cell lung cancer	Resistance to gefitinib, Proliferation, colony-formation ability, invasion	Differentiation grade, tumor invasion, TNM stage	hsa_circ_0004015/miR-1183/PDPK1
circ_0006528	Upregulated	oncogene	Breast cancer	Resistance to adriamycin, DNA synthesis, proliferation, invasion, migration, cell cycle, apoptosis	TNM stage, relapse-free survival, overall survival	circ_0006528/miR-7-5p/ Raf1/MEK/ERK
hsa_circ_0000504	Upregulated	/	Colorectal cancer	Resistance to 5-fluorouracil	/	hsa_circ_0000504/ hsa-miR-485-5p/STAT3
hsa_circ_0043632	Upregulated	/	Non-small cell lung cancer	Resistance to EGFR-TKI inhibitor AZD9291	/	hsa_circ_0043632/miR-492/ TIMP2
circ-MTO1	Downregulated	/	Breast cancer	Resistance to monastrol, proliferation	/	circ-MTO1/TRAF4/Eg5
hsa-circ-0001258	Downregulated	/	Osteosarcoma	Resistance to doxorubicin	/	hsa-circ-0001258/ hsa-miR-744-3p/GSTM2
CircPAN3	/	/	AML	Resistance to doxorubicin	/	circPAN3/miR-153-5p / miR-183-5p-XIAP

EGFR TKIs, as the first-line treatment for patients with EGFR mutations, includes gefitinib, erlotinib, and afatinib [160]. However, many patients developed resistance to EGFR TKIs within about 1–2 year after therapy, which largely limited the long-term efficacy of drug and was unfavorable for patients’ prognosis [161]. Recently, Zhou et al. reported that hsa_circ_0004015, a highly-expressed circRNA in NSCLC tissues, can act as a miR-1183 sponge to regulate 3-phosphoinositide dependent protein kinase 1 (PDPK1), thereby increasing the resistance of NSCLC cells to gefitinib [54]. Moreover, Chen et al. firstly revealed comprehensive analysis of circRNA profiling in EGFR-TKI inhibitor AZD9291-resistant NSCLC cells [162]. A total of 15,504 circRNAs were significantly dysregulated (With |fold change| ≥ 2 and *p* < 0.05), including 7966 upregulated and 7538 downregulated circRNAs [162]. It was predicted that hsa_circ_0043632 mediates NSCLC progression and AZD9291-resistance of NSCLC cells through miR-492/TIMP metallopeptidase inhibitor 2 (TIMP2) axis [162]. However, further research should be conducted to verify this prediction from bioinformatic analysis.

## Future prospective and conclusion

CircRNAs were initially thought to be functionless byproducts of aberrant RNA splicing [1]. The mystery of circRNAs has gradually been unveiled owing to the implication of high-throughput screening technology [39, 163]. A variety of circRNAs have been reported to mediate cancer occurrence and progression through various molecular mechanism, such as acting as miRNA sponges, interacting with RBPs, and regulating expression of parental genes [43, 82, 141].

Despite much advances in the research of circRNAs, there is still a long way ahead for circRNAs to be incorporated into clinical practice. Firstly, it is essential to develop a standard naming rule of circRNAs and perfect the building of the databases of circRNAs. The majority of circRNAs are named on the basis of their host genes or functions, and the condition is easily confused when several circRNAs arise from the same host gene or more than one circRNAs with relevant roles [64, 164]. Recently, Circbank, a comprehensive database, collects 140,790 human circRNAs with standard nomenclature, which can solve the conflicts caused by previous naming rules. Moreover, Circbank also introduces five critical features of circRNAs, including m6A modification of circRNAs, mutation of circRNAs, miRNA binding site, conservation of circRNAs, and protein-coding potential of circRNAs, giving superior to the design of CircBase, Circ2Traits, CircRNADb and CicrNet databases [165–169]. However, the addition of circRNAs of other species in Circbank dataset is in an urgent need [165].

For a century, the somatic mutation theory (SMT) has been the prevalent theory to explain carcinogenesis [170]. More recently, alternative theories have been introduced, such as tissue organization field theory (TOFT) [171]. The TME involves the coevolution of both cancerous cells and the surrounding stroma. The crosstalk between circRNAs and critical components of the TME can mediate tumourigenesis, angiogenesis, immune response, and metastatic progression. This new perspective on cancer research is different from the view of SMT that cancer is a cellular problem. The involvements of circRNAs in the TME may help us to rethink cancer progress and provide new approach for therapeutic use in cancer [16, 17, 118].

Moreover, larger tissue samples, longer follow-up visits, as well as the conduction of in-vivo assays are proposed to unveil the identification of circRNAs and improve the development of the molecular diagnosis. Exosomes have emerged as a novel approach for the treatment and diagnosis of cancer after RNA content was discovered in exosomes [172]. The stable expression of circRNAs in the exosomes and blood plasma may pave a new path for cancer diagnosis and treatment. However, considerable work is needed to solve the difficulties and defects of circRNA-based daignosis, such as high expense, existence of secondary structure, and limited knowledge of mechanism.

In this review, we briefly summarize the biogenesis, characteristics, classification and regulatory mechanism of circRNAs in various cancers. More importantly, we are first reviewing the interplay between circRNAs and key components of the TME, and further discussing their potential clinical value as biomarkers and the challenges of future research of circRNAs.

## Data Availability

Not applicable.

## Abbreviations

5-Fu: 5-Fluorouracil
ADM: Adriamycin
AGGF1: Angiogenic factor with g patch and fha domains 1
Ago2: Argonaut 2
CAEs: Cancer-associated endothelial cells
CAFs: Cancer-ssociated fibroblasts
CDK2: Cyclin dependent kinase 2
ceRNA: Competitive endogenous RNA
ChIP: Chromatin immunoprecipitation
CircRNAs: Circular RNAs
CIRI: CircRNA identification
ciRNAs: Circular intron RNAs
COL1A1: Collagen type I alpha 1 chain
CRC: Colorectal cancer
CSCs: Cancer stem cells
DXR: Doxorubicin
ecircRNAs: Exon circRNAs
eiciRNAs: Exon-intron circRNAs
EMT: Epithelial-to-mesenchymal
ERK: Extracellular signal-regulated kinase
ESCC: Esophageal squamous cell carcinoma
FGF: Fibroblast growth factor
GBM: Glioblastoma multiforme
GC: Gastric cancer
GSTM2: Glutathione S-transferase mu 2
HCC: Hepatocellular carcinoma
HEGF: Human epidermal growth factor
HIF1AN: Hypoxia-inducible factor 1-alpha inhibitor
HIF1α: Hyperpoic-inducible factor 1α
IRES: Internal ribosome entry site
lncRNA: Long non-coding RNAs
MDR: Multidrug response
ncRNAs: Non-coding RNAs
NSCLC: Non-small cell lung cancer
ORF: Open reading frame
OS: Osteosarcoma
PDK1: Pyruvate dehydrogenase kinase
PD-L1: Programmed death ligand 1
PDPK1: 3-phosphoinositide dependent protein kinase 1
PI3K: Phosphatidylinositol 3-kinase
PSS: Proximal splice site
PTEN: Phosphatase and tensin homolog
qRT-PCR: Quantitative reverse transcription polymerase chain reaction
RBP: RNA-binding protein
RNA-seq: RNA-sequencing
RNA-seq: RNA-sequencing
STAT3: Signal transducer and activator of transcription 3
TERT: Telomerase reverse transcriptase
TILs: Tumour-infifiltrating lymphocytes
TIMP2: TIMP metallopeptidase inhibitor 2
TME: Tumour microenvironment
TRAF4: Tumour necrosis factor receptor associated factor 4
TSP-1: Thrombospondin 1
VEGF: Vascular endothelial growth factor
